# Prevalence of human papillomavirus infection among Iranian women using COBAS HPV DNA testing

**DOI:** 10.1186/s13027-018-0178-5

**Published:** 2018-01-25

**Authors:** Farzane Jamdar, Farah Farzaneh, Fariba Navidpour, Sarang Younesi, Payam Balvayeh, Maryamsadat Hosseini, Robabeh Ghodssi-Ghasemabadi

**Affiliations:** 1 Preventive Gynecology Research Center (PGRC) ShahidBeheshti university of Medical science, Imam Hossein Hospital, Tehran, Iran; 2 Head of the preventive Gynecology Research Center (PGRC) ShahidBeheshti university of Medical science, Imam Hossein Hospital, Tehran, Iran; 3 Manager of post analytical quality control department in Nilou lab, Tehran, Iran; 4 Technical manager of nilou laboratory, Tehran, Iran; 5 Anatomical and clinical pathologist, Tehran, Iran; 6 Department of Biostatistics, Faculty of Medical Sciences, TarbiatModares University, Tehran, Iran

## Abstract

**Background:**

Persistent infection with High Risk Human Papillomavirus (HR HPV) typesplaysamajor role in the development of cervical cancer. Therefore, the detection of HR HPV types is an essential part of cervical cancer screening. The aim of this study was to estimate the prevalence of HR HPV infection among healthy women undergoing routine cervical cancer screening in Iran.

**Methods:**

In this cross-sectional study,the results of HPV DNA typing in 2453 normal Iranian womenwhowere referred for routine cervical cancer screening from September 2015 to March 2017 were analyzed. Participants were screened using COBAS assay for HPV DNA typing and liquid based cytology.

**Results:**

A total of 2453 healthy sexually active women were included in this study. The mean age was 35.1 ± 8.08 years. The overall prevalence of HR HPV infection was 10.3%. HPV16 was found in 73 (3%) women. The prevalence of HPV18 and other HR HPV typeswere 16(0.7%) and166 (8.2%),respectively. Approximately, 5% of the study population had an abnormal cervical cytology (ASCUS or worse), of whom 34% were infected by HR HPV.

**Conclusion:**

The prevalence of HR HPV infection among Iranian women has increased in the recent years which indicates the need for public education and health planning toprevent this cancer through vaccination and early diagnosis using screening tests.HPV DNA typing, diagnosisand the distribution of prevalent genotypes should be considered in the development of comprehensive cervical cancer prevention programs in Iran.

## Background

Cervical cancer is the third most common gynecologic cancer and the fourth leading cause of cancer death among women worldwide [1]. It has been demonstrated that persistent infection with high risk human papilloma virus (HR HPV) types playsa major role in the development of cervical intraepithelial neoplasia and cervical cancer [2–6]. Depending on the prevalence of HPV types in cervical cancer and its precursors, HPV types are classified as “low risk” and “high risk” [7]. Although HPV infection is the most common sexually transmitted disease in the world, themost detected HPV infections are transient and not expected to cause future high-grade cervical disease: 54% resolve spontaneously in one year and 91% in two years [8, 9]. Practically the prevalence and type-distribution of HPV infection vary between populations in different countries [10].

In the study by Khodakarami et al. 2012, the prevalence of high-risk HPV infection in healthy women was 5.1% in Tehran, Iran and the HPV 16 was the most common detected virus genotype (17). HPV 16 has been the most common type of infection in previous studies [11–17]. In the recent years,HPVs 31, 33, 45 and 58 have mostly been observed in many countries in East Asia [18]. HPV vaccination has been proven to offer near 100% protection against the development of cervical cancer and pre-cancerous lesion of HPV among individuals who have not previously been infected with HPV [11]. Based on these results, HPV DNA typing in clinical setting and the identification of HR-HPV genotypes is an important part of cervical cancer screening, as well as a means of providing valuable evidences necessary for the prevention and management of this cancer [11]. HPV DNA typing is not considered as routine practices of cervical cancer screening in Iran yet. The aim of this study wasto estimate the prevalence of HR HPV infection among Iranian healthy women undergoing routine cervical cancer screening using COBAS HPV typing assay. The results of the current study might help us in making public health decisions regarding the cervical cancer screening and HPV vaccination to prevent and decrease the incidence of thiscancer in Iran.

## Methods

### Study population

In this cross-sectional study, the result of the HPV screening of 2453healthy womenwho were referred toNiloulaboratory, Tehran, Iranfor cervicalcancer routine screening from September 2015 to March 2017 were analyzed. Demographic data, cervical cytology reports, and HPV test results were collected. Demographic data were recorded by trained laboratory staff. Cervical smears were obtained from the cervix of each participant with cyto-brush for liquid-based cytological analysis and HPV DNA testing bygynecologists. Cervical cytology smears were read by acytopathologist who was unaware of participants’ HPV DNA test results.After providing HPV DNA test and cytologyresults, women with ASCUS or worse or positive HR HPV DNA were referred to a gyneconcologist for colposcopy examination.

### HPV genotyping

Roche cobas® HPV test (Roche Molecular Systems, Pleasanton CA), which is one of four approved HPV tests by the US Food and Drug Administration was used for HPV DNA typing. The COBAS® HPV test can detect 14 high risk HPVs. This HPV DNA testing method separatelydetects HPV16 and HPV18and a pool of 12 other high-risk (HR) HPVs types together (31,33,35,39,45,51,52,56,58,59,66,68) [19, 20].

After obtaining smeared cell slides for ThinPrep liquid-based cytology test, the remaining cell samples on the cytobrush were stored at room temperature for further analysis (HPV DNA is stable for 4 months). The HPV detection and pathological diagnosis were performed independently in two departments. Roche COBAS® HPV test is an automated qualitative in vitro test for the detection of human papilloma virus (HPV) DNA in patient specimens. The test utilizes amplification of target DNA by the polymerase Chain reaction (PCR) and nucleic acid hybridization for detection of 14 high-risk HPV (hr-HPV) types in a single analysis. The test specifically identifies HPV16 and HPV18 while concurrently detecting the rest of the high risk types (31,33,35,39,45,51,52,56,58,59,66, and 68) at clinically relevant infection levels.

### Statistical analysis

Continuous data were represented by mean and standard deviation, count and proportion was applied to show categorical data. The distribution of categorical data in different groups was tested using chi-squared test. Proportions were reported with 95% confidence interval. The trends of proportions were tested using Cochrane-Armitage test. The significance level was set at 0.05 level and SPSS software version 21 was used for analysis.

The study was approved by thePreventative Gynecology Research Center (PGRC), ShahidBeheshti University of Medical Sciencesethics committee. All of the participants were informed about the study and signed the written consent form.

## Results

A total of 2453 healthy sexuallyactive women were included in this study. The mean age was 35.1 ± 8.08 years. The overall prevalence of HR HPV infection was 10.3% (253 women).

**Fig. 1:**
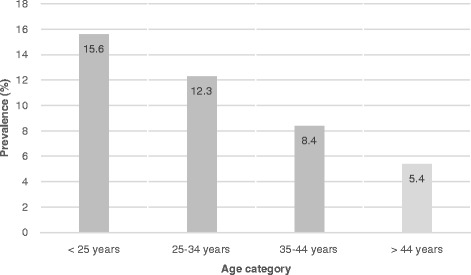
HPV prevalence based on age categories

HPV16 infection (alone or with other HR HPVs) was detected in 73 (3%) women. HPV18 infection (alone or with other HR HPV) was detected in 0.7% of participants,other 12 HR HPV types (31,33,35,39,45,51,52,56,58,59,66,68) were detected in 8.2% of womenaccording to COBAS HPV DNA assay. The prevalence of HPVgenotype is summarized in Table 1. There was a statistically significant decrease in the HPV prevalence with age categories (*P* < 0.001). Results are shown in Fig. 1.

**Table 1:** HPV genotype results

HPV TYPING	Frequency	Prevalence	95%CI for Prevalence
HPV 16 (single infection)	44 (1.8)	1.8%	(1.3% - 2.4%)
HPV 18 (single infection)	10 (0.4)	0.4%	(0.21% - 0.78%)
HPV 16& HPV18 as co-infections	0	0%	–
Other HR HPVs (31,33,35,39,45,51,52,56,58,59,66,68)	166 (6.8)	6.8%	(5.8% - 7.9%)
Other HR + 16	27 (1.1)	1.1%	(0.74% - 1.6%)
Other HR + 18	4 (0.2)	0.2%	(0.05% - 0.45%)
Other HR + 16 + 18	2 (0.1)	0.1%	(0.01% - 0.33%)
Sum*	253 of 2453(10.3)	10.3%	(9.2% - 11.6%)
Total	2453 (100)	100%	–

*Sum of the HPV typing

HPV: Human Papilloma Virus, HR: High Risk

Cervical cytology results of 793 women were available. The mean age of women with known and unknown cervical cytology results were 34.37 ± 7.51 and 35.44 ± 8.31, respectively.

Majority of them with known cervical cytology results [95 % (753 out of 793)] had normal pap-smear. The prevalence of atypical squamous cells of undetermined significance (ASCUS), low-grade squamous intraepithelial lesion (LSIL), high-grade squamous intraepithelial lesion (HSIL), and atypical squamous cells cannot exclude high grade SIL (ASC-H) were (3%), (1.9%), (0%), and (0.1%), respectively in this population.HR HPV DNA was detected in 13.5% (102 out of 753) of women with normal cytology.The prevalence of HR HPV was significantly higher in patient with abnormal cervical cytology than women with normal cervical cytology (34% versus 13.3%, *P* < 0.001). The prevalence of HR HPV infection increased with greater diagnosis severity. The prevalence of HR HPV infection was 54.2% in ASCUS and 66.7% in LSIL. The results of cervical cytology are presented in Table 2 for positive and negative findings of HPV. Other 12 HR HPV types were predominant types of infection in all grades of both abnormal and normal cytology. The distribution HPV genotype are shown in Table 3 based on cytology results.

**Table 2:** Prevalence of HPV based on cytology

		HPV	
PAP smear report	N(%)	Negative N(%)	Positive N(%)
Normal	753(95)	651 (86.5)	102 (13.5)
ASCUS	24 (3)	11 (45.8)	13 (54.2)
LSIL	15 (1.9)	5 (33.3)	10 (66.6)
HSIL	0 (0)	0	0
ASCH	1 (0.1)	1 (100)	0
Total	793 (100)	668 (84.2)	125 (15.8)

ASCUS: Atypical squamous cells of undetermined significance,LSIL:Low-Grade Squamous Intraepithelial Lesion,HSIL:High-Grade Squamous Intraepithelial Lesion,ASCH:Atypicalsquamouscells cannot excluded high grade SIL

**Table 3:** Distribution of HPV genotype based on cytology results

	HPV 16	HPV 18	HPV HR	16 + HR	16 + 18 + HR	18 + 16	18 + HR
Normal	17 (16.7%)	2 (2%)	71 (69.5%)	11 (10.8)	1 1%	0	0
ASCUS	2 (15.4%)	0	8 (61.5%)	3 (23.1%)	0	0	0
LSIL	3 (30%)	0	5 (50%)	1 (10%)	0	0	1 (10%)

ASCUS: Atypical squamous cells of undetermined significance,LSIL:Low-Grade Squamous Intraepithelial Lesion,HSIL:High-Grade Squamous Intraepithelial Lesion,ASCH:Atypicalsquamouscells cannot excluded high grade SILl

## Discussion

In this study the overall prevalence of HR HPV infection was 10.3% using COBAS HPV DNA typing assay among healthy women subjected to routine cervical cancer screening.Approximately, 5% of available cervical cytology results were abnormal (ASCUS or worse).the prevalence of HPV infection decreased significantly with age. The highest prevalence among individuals under the age of 25 was 15.6%, which gradually decreased and reached 4.5% among women over the age of 44 years. Zandi et al. (2008) reported that the prevalence of HPV infection in women undergoing routine pap smears was 5.5% in Bushehr, Iran [21]. Moreover, Khodakarami et al. (2012) reported the 7.8% HPV infection prevalence in Tehran using GP5+/6+ PCR-based assay, but in their report, the prevalence of HR HPV infection was 5.1% with no significant variation by age [17]. The prevalence of abnormal papsmear was 4.1% in their study and HPV16 was the most prevalent genotype [17]. The study of Khodakarami et al. (2012) on 18- to 59-year-old women in Tehran showed that the prevalence of HPV infection was significantly higher among women in polygamous marriages, divorced women, and those reporting husband’s absence from home for more than 7 nights/month [17]. In another study by Yousefzadeh et al. (2013), the prevalence of HPV infection in women was 31.1%in Tehran, Iran. The reason for this rate ofHPVprevalencein this study was that they examined the prevalence of low-risk and high-risk HPVs together and the studied population was not general population, but were those who were examined for gynecologicalsymptoms [22]. In this study, we used COBAS method to detect HPV infection, which is approved by FDA. The approval is based on ATHENA study which was performed on more than 47,000 women in the US [23]. Based on the ATHENA study, COBAS is the only FDA approved method for primary cervicalcancer screening [23, 24]. In the ATHENA study, it was found that primary screening of HPV in women ≥25 years is as effective as dual screening in conjunction with cytology, but initial screening with HPV alone requires less testing [24].

The prevalence of HPV infection and its distribution differed by age, geographic region, and cytology findings [11]. Wu EQ et al. (2013), andZhao (2015) reported 14.3% and15.5%, respectively for the prevalence of HPVinfection amongChinese women [11, 12]. Wuet al. (2017) reported 22.7% and 17.3% for the prevalence of any HPV infections and oncogenic HPV type, respectively in China [25].

The prevalence of HPV infection was 19% among Portuguese women in (2011) [26]. Furthermore, the prevalence of high risk HPV was reported 8% in Italy by Giorgi et al. (2012) [15]. While, based on a study by Monsonegoet et al. (2012), onwomenin Paris, the overall prevalence of HPV infection differedusingAPTIMA HPV assay (AHPV) (10.1%) and Hybrid capturer 2 (HC2)(15%)assay [14].McQuillan, et al. (2017) reported that the prevalence of 39.9% for any and 20.4% for high risk HPVsamong American womenaged 18–59 years [27].

The prevalenceofHR HPV infection among Iranian women was more than Italian women but it was less thanAmerican, Chinese and European (Paris and Portugal) records. Compared withkhodakarami et al. 2012, and Zandi et al. 2008, we have been confronting a rising rate of HPV infection in our country since 2008. It should be emphasized that the HPV DNA detection was carried outusing different methods in these studies. Moreover, in our study, the rate of abnormal pap-smear (5%) increased in comparison with the rate (4.1%) reported by khodakarami et al. 2012.

An increase in the prevalence of HPV infection in recent years has multiple causes. Iran’s population is predominantly young, and we are witnessing a change in the sexual behavior of teenagers compared to the past.These behavioral changes and lack of adequate education about sexual issues have led to a high rate of referral to genecology clinics due to genital wart and the complications of HPV infection. Another cause of increased prevalence of HPV is the prevalent use of hookah among teenagers and young people. This type of tobacco has traditionally been used in Iran. Also, the community is familiar with the dangers and disadvantages of smoking, but considering the history of smoking hookah in Iran, most people consider this as a safe form of tobacco and are not aware of its dangers.

On the other hand, vaccination against HPV infection is not currently routine in Iran and the cost of the vaccine is not covered by the insurance companies either. Also, the study of Jalilvand et al. (2014) showed that in addition to cervical cancers, HPV type 16 and 18 are also the most common types of viruses in other HPV- induced cancers (such as head and neck SSCs). Based on this, the existing vaccines, in addition to reducing the cervical cancer, lead to decreased in other HPV-dependent cancers [28]. Providing the statistics on the high-risk HPVprevalence in Iran willassist us in designingaccurate health educational programs to reduce the prevalence of HPV infection and cervical cancer and to diagnosethe disease in early stages in our country in the coming years.

The limitation of the current study are as follows:

First, the COBAS HPV testing is organized for clinical decision making in order to manage cervical lesions. This method reports HPV16, 18, and 12 other high risk types of HR HPV together. Therefore, we were not able to separately find out the prevalence of other 12 types of HR HPV. While, knowing the common genotypes of HPV infection has clinical significance for vaccination.Second, the HPV analysis is carried out at NILOU laboratory using the COBAS HPV DNA testing and many laboratories from different cities of Iran analyzed cytological specimens themselves and just referred the samples to NILOUlaboratory for HPV typing using the COBAS assay. Therefore, pap-smear results of many patients were not available.

## Conclusion

The results of the current study showed that the prevalence of HPV infection in Iran is close to the worldwide HPV prevalenceandit might increase. Therefore, all suggested preventative issues (education on sexual activity, social behavior, vaccination and screening programs)by international health organizations (i.e. WHO) regarding HR HPV could be consideredfor Iranian population by health workers.The present study is the first study in Iran to investigate the prevalence of HPV using the COBAS method.
